# The perfusion index of healthy term infants during transition at birth

**DOI:** 10.1007/s00431-015-2650-1

**Published:** 2015-10-24

**Authors:** Jacco K. Kroese, Jeroen J. van Vonderen, Ilona C. Narayen, Frans J. Walther, Stuart Hooper, Arjan B. te Pas

**Affiliations:** Department of Pediatrics, Division of Neonatology, Leiden University Medical Center, J6-S, PO Box 9600, 2300 RC Leiden, The Netherlands; MIMR-PHI Institute for Medical Research, Monash University, Clayton, Victoria Australia

**Keywords:** Neonatal transition, Newborn, Perfusion index, Heart rate, Oxygen saturation

## Abstract

Perfusion index is a continuous parameter provided by pulse oximetry and might be useful for evaluating hemodynamic changes at birth and identifying transitional problems. The objective was to describe perfusion index values in term infants immediately after birth. Perfusion index of 71 healthy term born infants were recorded during the first 10 min after birth, using a pulse oximetry sensor placed preductally. A Wilcoxon signed-rank test was used to compare between time points. No significant trend in perfusion index could be observed in term-delivered infants. There was a significant difference between 2 and 3 min (2.4 (1.6–5.0) vs. 2.3 (1.6–3.7), *p* = 0.05) and between 3 and 4 min after birth (2.3 (1.6–3.7) vs. 2.1 (1.4–3.2), *p* < 0.001). There was no significant change in median PI values in the following 8 min.

*Conclusion*: Perfusion index does not change significantly during transition at birth in healthy term infants born by normal vaginal delivery or cesarean section. Large variation in perfusion index causes monitoring this parameter to have limited value.

**Table:** 

**What is known:**
• *Perfusion index is a non*-*invasive indicator for peripheral perfusion*. • *Perfusion index values* <*1.24 are seen as an accurate predictor for severity of illness for infants admitted to the neonatal intensive care unit*.
**What is new:**
• *Although significant physiological changes occur during birth, perfusion index remains stable*. • *Large variation in perfusion index causes monitoring of this value to have limited value as an additional parameter for evaluating transition at birth*.

## Introduction

In recent years, pulse oximetry (PO) has been recognized as an easily applicable, non-invasive monitoring tool and is now routinely used in the delivery room to monitor transition at birth [5, 15, 22, 24]. PO is recommended for objective evaluation of heart rate (HR) and oxygen saturation (SpO_2_) at birth [5, 26], and it is used to decide if interventions are necessary [16, 20, 31]. In addition to HR and SpO_2_, the infrared signal of a PO can also be used to determine the perfusion index (PI). PI is calculated as the ratio of the pulsatile signal (arterial blood flow) indexed against the non-pulsatile signal (static blood flow, skin, and other tissues) and is a non-invasive indicator for peripheral perfusion [17]. PI has been related to the flow through the superior vena cava in preterm infants [28] and volume responsiveness in neonates [2]. Moreover, low PI values (<1.24) has been identified as an indicator for severe illness in newborn infants [10, 12].

During neonatal transition, significant respiratory and hemodynamic changes occur, influencing cardiac output as well as systemic and peripheral perfusion [3, 4, 19, 25]. Currently, only SpO_2_ and HR are used for clinical evaluation, but these parameters do not reflect the complete hemodynamic status of the infant at birth [30]. Tissue perfusion is not only dependent on HR but also stroke volume and blood pressure [29]. Continuous monitoring of the perfusion by means of PI measurements could be valuable for evaluation of newborn infants during neonatal transition as large changes take place during the neonatal transition. Although PI values have been described in term infants during neonatal transition [9, 14] and the first days after birth [1, 12, 13], PI values obtained immediately after birth have not yet been reported. The objective of this study was to evaluate and to establish reference values for PI of healthy term-born infants during the first 10 min after birth.

## Methods

We analyzed PO recordings of healthy term infants born via uncomplicated vaginal delivery or elective cesarean section who did not need medical support. The study was approved by the LUMC institutional review board and the parents were approached before birth for informed consent. Recordings of term infants born between February 2012 and March 2013 were reviewed retrospectively. Gestational age was determined by performing an antenatal ultrasound, which is standard of care in the Netherlands. Recordings were included if PI measurements were obtained shortly after birth and time of birth was indicated. Recordings were excluded if infants received respiratory support.

Directly after birth, the infant was placed under a radiant warmer. PI, HR, and SpO_2_ were recorded by one member of the research team. During each delivery, a pediatric resident, a neonatal IC nurse, and one researcher were present. The researcher (JV) put the PO sensor on. The PO sensor (M-LNCS NeoPt-500, Masimo SET, Masimo, Irvine, CA, USA) was always placed on the ulnar aspect of the right wrist providing preductal arterial values [20] and connected to a pulse oximeter (Masimo Radical 7, Masimo, Irvine, CA, USA) [21]. PI, HR and SpO_2_ were recorded at maximum sensitivity every 2 s from 2 min after birth until 10 min after birth or earlier if the infant was placed in the transport incubator. The recorded values were collected using the Spectra Physiological Recording Program (Grove Medical, London, UK) and stored on a laptop. Data were averaged for each infant at each 60 s interval, comprising of the data points of 10 s of data before and after each 60 s interval. Data were considered valid for analysis if PI, HR, and SpO_2_ were simultaneously present at a time point and the plethysmography pulse wave was confirmed to be artifact free. Limits of PI values identified to be unvalid were ≤0.02 and ≥20 and simultaneous absence of HR and SpO_2_ values [18].

### Statistical analysis

Data were analyzed using SPSS 20.0 for Windows (IBM, Chicago, IL, USA). All variables were tested for normality using the Kolmogorov-Smirnov test. Data were presented as mean (SD) or median (IQR), where appropriate. For non-normally distributed data, a Wilcoxon signed-rank test was used to compare PI between time points within groups. To account for multiple comparisons between the time points, tests were performed using the Bonferroni corrected level (*p* = 0.05/2 = 0.025). A (two-sided) *p* value of <0.05 was considered as statistically significant.

## Results

PO values of 71 infants with a mean (SD) gestational age of 40 (1) weeks and a birth weight of 3575 (482) grams were recorded and analyzed. Thirty-nine infants were born after cesarean section and 32 infants were born after vaginal delivery. Median (IQR) Apgar score was 9 (9–9) at 1 min and 10 (10–10) at 5 min. 97 % of data (5860 data points) of the PI measurements were of good quality and were included.

**Fig. 1:**
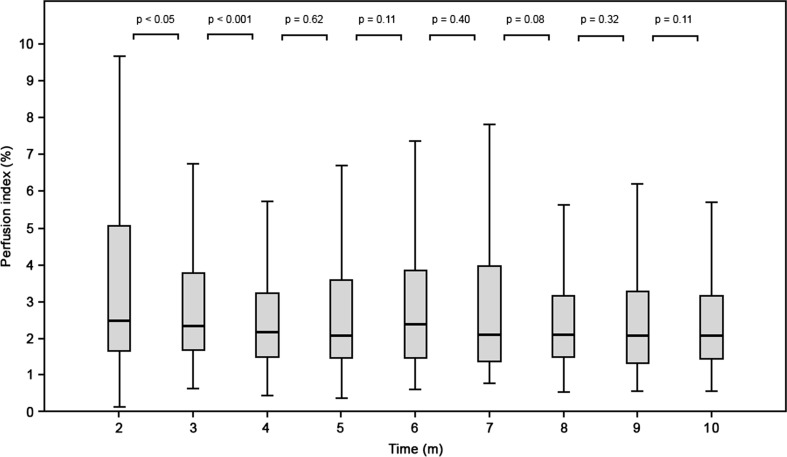
Median (IQR) of perfusion index (PI) values of healthy term born term infants during the first 10 min of life

The median time (IQR) taken to acquire PI, HR, and SpO_2_ simultaneously was 110 s (110–126 s). HR and SpO_2_ were within ranges described by Dawson et al. (Table 1) [6, 8]. The median PI over the first 10 min was 2.1 (1.4–3.5). There was no significant difference between infants born by cesarean section or after vaginal delivery. There was a significant difference between 2 and 3 min (2.4 (1.6–5.0) vs. 2.3 (1.6–3.7), p = 0.05) and between 3 and 4 min after birth (2.3 (1.6–3.7) vs. 2.1 (1.4–3.2), *p* < 0.001). There was no significant change in median PI values in the following 8 min (Fig. 1).

**Table 1:** Perfusion index, heart rate, and oxygen saturation measurements (median (IQR)) of healthy term infants during the first 10 min after birth

Time (min)	*N*	PI (%)	HR (bpm)	SpO_2_ (%)
2	54	2.4 (1.6–5.0)	132 (64–165)	82 (73–90)
3	62	2.3 (1.6–3.7)	147 (105–165)	85 (76–91)
4	62	2.1 (1.4–3.2)	145 (124–157)	85 (80–91)
5	64	2.0 (1.4–3.5)	141 (128–151)	88 (79–93)
6	65	2.3 (1.4–3.8)	144 (124–154)	89 (82–93)
7	68	2.0 (1.3–3.9)	144 (126–156)	90 (86–94)
8	69	2.0 (1.4–3.1)	142 (125–156)	92 (87–95)
9	69	2.0 (1.3–3.2)	142 (129–152)	92 (88–96)
10	61	2.0 (1.4–3.1)	144 (133–151)	93 (89–96)

*PI* perfusion index, *HR* heart rate, *SpO*_*2*_ oxygen saturation

## Discussion

This is the first study reporting the course of PI of healthy term infants during transition at birth. Significant physiological changes occur during transition and although this is reflected by changes in SpO_2_ and HR, we observed that PI decreases significantly during between 2 and 4 min after birth, after which remains stable, and no trend could be observed. Currently, the only non-invasive monitoring used in the delivery room is the measurement of heart rate. This does not reflect the complete hemodynamic status of the infant at birth [30]. PI could be of added value; however, the large variation in PI causes monitoring of PI to have limited value as an additional parameter for evaluating transition at birth.

Hawkes et al. found a moderate correlation with HR and found no significant correlation for PI with blood pressure values, lactate levels, and gestational age. Furthermore, no trend was observed during the first 10 min of life [14].

The observed PI is similar to a previous study by de Felice et al., reporting no differences in PI values measured at 0–1 min and 1–5 min after birth [9]. This could indicate that pulsed waveforms measured in preductal arteries remain stable despite the large hemodynamic changes that occur during transition. All infants cried and most likely aerated their lungs in the first breaths after birth before the cord was clamped. It has been shown that breathing before cord clamping leads to more stable changes in cardiac output during transition, which could have influenced the measured PI [19, 27]. After cord clamping, systemic vascular resistance increases when the lungs are aerated and pulmonary vascular resistance decreases, which causes a left-to-right ductal shunt [4]. This could have mitigated changes in peripheral circulation, and is in line with our previous observation that peripheral blood pressure remains stable during transition [30]. More studies on circulation in the extremities would be needed to confirm or refute this.

We measured a lower preductal PI at 5 min after birth than de Felice et al. measured postductally in healthy term infants (4.4 (2.1) vs. 2.0 (1.4–3.5)) [9]. This difference is difficult to explain, as we expect postductal perfusion to be maintained secondary to an increase in left-to-right ductus arteriosus shunting [32]. However, as PI is a scaled numerical value derived from the magnitude of the pulsations, it is possible that a ductal steal (increase in left-to-right shunt in the ductus arteriosus) might lead to larger pulse waveforms postductally, resulting from a lower end-diastolic pressure. This is similar to the presence of bounding pulses as a clinical sign for a patent ductus arteriosus.

De Felice et al. suggested PI values <1.24 as an accurate predictor for severity of illness [9]. Although we have not compared the values of the healthy term cohort with sick infants at birth, we cannot confirm this cutoff value as approximately 25 % of our healthy term infants had a PI <1.24, and none of these infants needed medical support or were admitted for medical care later on. Also, approximately 25 % of the infants had a PI above 3.5 during the same time points. This difference could be explained by various individual differences such as local vasodilatation and vasoconstriction [7].

The nature of this study was retrospective. We intended to provide PI values for healthy infants during the first minutes after birth and infants that needed medical support were excluded. Therefore, this did not influence our findings. A limitation of our study is the performance of the PO which could explain the wide range in PI observed during transition [11]. Whether some of this variability might be device- specific remains unclear, as no studies have yet been performed comparing PI using different PO monitors [23]. In addition, PI varies between individuals reflecting changes in the physiologic state at the measurement site [7]. Although we did not find a clear trend as there was large variability, comparable to HR and SpO_2_, a continuous monitoring could give the caregiver more information than a single value. Although we reported values of healthy term infants, comparison with term infants with transitional problems is needed to determine whether the PI is a useful parameter for hemodynamic evaluation of transition and clinical decision making.

In conclusion, we reported PI measurements in healthy term infants during the first 10 min after birth. There was a small but significant decrease in PI during the first 4 min, after which PI remained equal and variability was high healthy term born infants.

## Abbreviations

HR: Heart rate
PI: Perfusion index
PO: Pulse oximetry
SpO_2_: Oxygen saturation
